# Importations of COVID-19 into African countries and risk of onward spread

**DOI:** 10.1186/s12879-020-05323-w

**Published:** 2020-08-13

**Authors:** Haoyang Sun, Borame L. Dickens, Alex R. Cook, Hannah E. Clapham

**Affiliations:** Saw Swee Hock School of Public Health, National University of Singapore, 12 Science Drive 2, Singapore, 117549 Republic of Singapore

**Keywords:** Coronavirus, COVID-19, SARS-CoV-2, Africa, Mathematical modelling

## Abstract

**Background:**

The emergence of a novel coronavirus (SARS-CoV-2) in Wuhan, China, at the end of 2019 has caused widespread transmission around the world. As new epicentres in Europe and America have arisen, of particular concern is the increased number of imported coronavirus disease 2019 (COVID-19) cases in Africa, where the impact of the pandemic could be more severe. We aim to estimate the number of COVID-19 cases imported from 12 major epicentres in Europe and America to each African country, as well as the probability of reaching 10,000 cases in total by the end of March, April, May, and June following viral introduction.

**Methods:**

We used the reported number of cases imported from the 12 major epicentres in Europe and America to Singapore, as well as flight data, to estimate the number of imported cases in each African country. Under the assumption that Singapore has detected all the imported cases, the estimates for Africa were thus conservative. We then propagated the uncertainty in the imported case count estimates to simulate the onward spread of the virus, until 10,000 cases are reached or the end of June, whichever is earlier. Specifically, 1,000 simulations were run separately under four different combinations of parameter values to test the sensitivity of our results.

**Results:**

We estimated Morocco, Algeria, South Africa, Egypt, Tunisia, and Nigeria as having the largest number of COVID-19 cases imported from the 12 major epicentres. Based on our 1,000 simulation runs, Morocco and Algeria’s estimated probability of reaching 10,000 cases by end of March was close to 100% under all scenarios. In particular, we identified countries with less than 1,000 cases in total reported by end of June whilst the estimated probability of reaching 10,000 cases by then was higher than 50% even under the most optimistic scenario.

**Conclusions:**

Our study highlights particular countries that are likely to reach (or have reached) 10,000 cases far earlier than the reported data suggest, calling for the prioritization of resources to mitigate the further spread of the epidemic.

## Background

In late December 2019, a novel coronavirus (SARS-CoV-2) was identified among patients presenting with viral pneumonia in Wuhan city, China [1]. Since then the number of coronavirus disease 2019 (COVID-19) cases and deaths increased rapidly [2, 3], and the city was locked down by the Chinese government on 23^rd^ January 2020. By late February, there had only been limited importations from and to places outside China [4]. However, new epicentres in Europe and America emerged shortly thereafter, causing a second wave of importations that further accelerated the spread of the pandemic [4]. Most countries have since then imposed travel restrictions to prevent further importation of COVID-19 cases [5]. By 15^th^ July 2020, over 13 million cases and 574,000 deaths had been confirmed worldwide [4].

A particular area of focus has been on countries in Africa, with worries about missed imported cases and what the impact will be of widespread transmission given the other heavy health burdens in these countries. The first confirmed case in Africa was reported in Egypt on 14^th^ February 2020, and two weeks later, the virus was found in sub-Saharan Africa with a reported case in Nigeria [4]. By 15^th^ July, over 600,000 cases had been reported in the whole of Africa, with substantial variation in the reported cumulative incidence across different countries [4]. This inter-country heterogeneity can be due to a wide range of factors, such as the number of imported cases, the capacity to conduct tests for COVID-19, surveillance efforts, as well as travel and movement restrictions which vary widely from country to country depending on the local context [5]. The reported data alone thus do not provide a clear depiction of the outbreak situation especially in countries with very limited surveillance capacities, and additional studies are needed to narrow the knowledge gap between the reported data and the real disease burdens.

Previous work has estimated the risk of importation from China at the early stage of the pandemic [6], assessed each African country’s capacity to respond to outbreaks [6], systematically collated information on the importation events reported by the sub-Saharan countries [7], and projected the spread of the epidemic seeded by the early cases represented in the World Health Organization Situation Reports [8]. It is still unclear how many cases may have been introduced to Africa from the new epicentres in Europe and America, although the reported case data do suggest that the size of this second wave of importations has been much larger than the first wave of importations from China [7]. In this study, we aim to estimate the number of COVID-19 cases imported from the major epicentres in Europe and America, and the magnitude of onward spread in each African country. This method is insensitive to the different testing and reporting systems that are in place in different countries.

## Methods

### Data

#### Case data

We collated data on the daily number of imported cases in Singapore reported by 31^st^ March from the following 12 epicentres: Austria, Belgium, France, Germany, Italy, Netherlands, Portugal, Spain, Switzerland, Turkey, United Kingdom, and United States, which accounted for over 90% of Singapore’s reported number of imported cases from countries outside of Asia [9]. These data will be used later to estimate the number of imported cases in Africa. In addition, we obtained the number of imported cases reported by each sub-Saharan African country by 31^st^ March [7], as well as the total number of confirmed cases in each African country by end of March, April, May, and June respectively [4].

#### Government response data

For each country, we collated the date on which each of the following policies came into force: [1] banning non-citizens and non-residents from entry (the start date could vary depending on the epicentre country from which a visitor arrived) [2]; mandatory (self-) quarantine for travellers arriving from each of the 12 epicentre countries mentioned earlier [3]; Stay-at-home order for all non-essential workers (hereinafter referred to as “stay-at-home order”). We reviewed the following sources: [1] country-level internal and international restrictions collated by the International SOS [5], [2] Oxford COVID-19 Government Response Tracker [10], [3] international travel restrictions collated by the International Air Transport Association [11], as well as [4] Wikipedia, where a separate page was available for each country containing information regarding the government response. For each Wikipedia page, we manually reviewed the online reports listed in the references to exclude data with unconfirmed or unreliable sources. If stay-at-home order came into force in different states of the same country at different times, only the earliest date was recorded. Since late April, some countries have lifted (and in rare cases, re-imposed) stay-at-home order, and the corresponding dates were taken from the Oxford COVID-19 Government Response Tracker database [10]. The government response data used in this study have been included within the Additional file 1.

#### Travel data

We obtained the total number of air ticket bookings for each origin-destination route allowing for up to two connections during March 2017 from the Official Airline Guide. This will be used later to estimate the ratio of air passenger volumes between pairs of origin and destination countries, which we assumed to be relatively stable over time.

### Statistical analyses

#### Estimating the number of imported cases

For each African country *r*, we denote the daily number of air passengers that arrived from an epicentre country *e* by${v}_{e\to r}^{(t)}\ \left(t={t}_e,\kern0.5em {t}_e+1,\dots, {T}_{e\to r}\right)$, where *t*_*e*_ refers to the start date of the COVID-19 epidemic in the epicentre country *e*, and *T*_*e* → *r*_ refers to the last day that non-citizens and non-residents travelling from country *e* were allowed to enter country *r*. Each day the probability that an air passenger travelling from country *e* to country *r* was an imported case is denoted by${p}_e^{(t)}$, which we assume to be dependent on both the origin country *e* and time *t*, but independent from the destination country *r*. In other words, the destination location was assumed to have a negligible impact on the risk of a traveller being an imported case, controlling for the origin location and travel date. Hence, the total number of COVID-19 cases imported from an epicentre country *e* to an African country *r* by the time the travel ban came into force (denoted by *M*_*e* → *r*_ below) can be approximated using a Poisson distribution (Refer to the Additional file 2 for the derivation details):

$$ {M}_{e\to r}\ \dot{\sim}\  Po\left(\sum \limits_{t={t}_e}^{T_{e\to r}}{v}_{e\to r}^{(t)}\bullet {p}_e^{(t)}\right). $$

We used the imported COVID-19 case data reported by Singapore as well as flight data to provide a conservative estimate for *M*_*e* → *r*_, under the assumption that Singapore, being one of the countries with the highest surveillance capacity [12], has detected all the imported cases. Owing to the delay from infection to hospital admission, we considered all cases imported from country *e* to Singapore that were *reported* by date (*T*_*e* → *r*_ + 9) (hereinafter denoted as *SG*_*e*, *r*_) based on Linton et al.’s estimated mean incubation period and time from illness onset to hospital admission [13]. We assumed that the ratio between the daily number of air travellers from epicentre *e* to country *r* and to Singapore remained stable in the presence of the changes in flight pattern in response to the COVID-19 pandemic (i.e. the percentage change in air passenger volumes was consistent across the two country pairs). This allows us to model *M*_*e* → *r*_ (and *SG*_*e*, *r*_) as Poisson random variables with mean parameters proportional to the numbers of air passengers travelling from epicentre *e* to country *r* (and to Singapore) using the March 2017 flight data (Refer to the Additional file 2 for the derivation details):

$$ {\displaystyle \begin{array}{c}{M}_{e\to r}\dot{\sim}\  Po\left({\beta}_{e,r}\bullet {\sum}_{t\ \mathrm{in}\ \mathrm{Mar}\ 17}{v}_{e\to r}^{(t)}\right),\\ {}{SG}_{e,r}\dot{\sim}\  Po\left({\beta}_{e,r}\bullet {\sum}_{t\ \mathrm{in}\ \mathrm{Mar}\ 17}{v}_{e\to SG}^{(t)}\right).\end{array}} $$

Here, *β*_*e*, *r*_ refers to the proportionality constant to be estimated using the reported value of *SG*_*e*, *r*_ and flight data, and was assigned a uniform prior with support (0, 1). We performed Markov Chain Monte Carlo to sample from the posterior distribution of *β*_*e*, *r*_ using the JAGS software [14]. A total of 10 chains were run in parallel, each with 2,000 iterations burn-in and 15,000 iterations thinned and subsequently merged to obtain a posterior sample of size 5,000. Both Geweke’s statistic and Brooks & Gelman’s potential scale reduction factors were derived to assess convergence (Refer to the Additional file 2 for more details) [15, 16]. The posterior sample for all the model parameters was then used to estimate the uncertainty distribution of the total number of COVID-19 cases imported from the 12 major epicentres to each country.

In March 2020, a spike in the number of cases imported from United Kingdom and United States was observed in Singapore, which was partly due to the increase in the number of returning Singaporean students studying overseas [17]. This change in flight patterns, however, may not be applicable to all African countries. Therefore, to be even more conservative, we also derived the imported case count estimates excluding United Kingdom and United States from the 12 epicentre countries previously considered. The resulting estimates were subsequently used in the simulations of the onward spread of SARS-CoV-2 to get our estimates of case numbers over time.

#### Simulating the onward transmission following importation

**Fig. 1:**
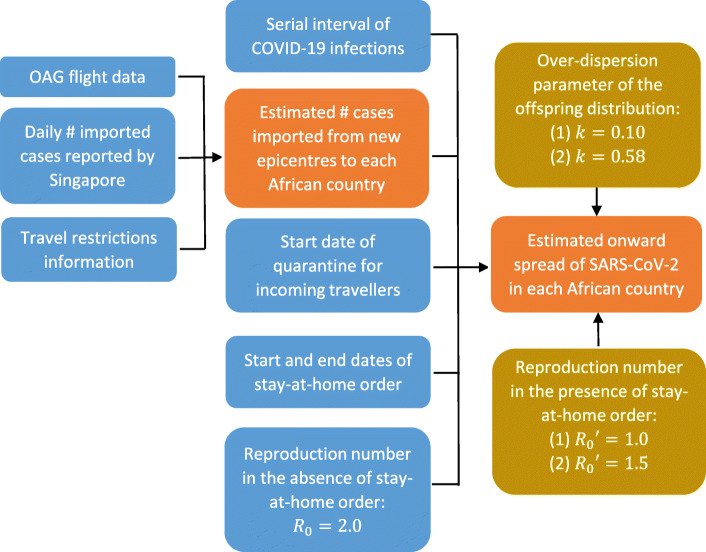
Schematic overview of the methods. Blue boxes denote input data, and orange boxes output estimates. Brown boxes are used to show the model parameters for which more than one possible value was specified to test the sensitivity of our simulation results.

We performed 1,000 simulations drawing from our estimated distribution of the number of imported cases to project the onward spread of SARS-CoV-2 in each country up to 30^th^ June 2020 or the date when we estimate 10,000 cases was reached, whichever was earlier. The time of infection for the cases imported from country *e* to country *r* was simulated via resampling from the reporting dates of the *SG*_*e*, *r*_ cases, which was then shifted backwards by 9 days to account for the delay from infection to hospital admission based on Linton et al.’s estimates [13]. To account for the effect of quarantine measures on the onward transmission, we only included the estimated imported cases who had acquired the infection prior to the mandatory quarantine of travellers coming into force, so that the estimation of local SARS-CoV-2 spread is conservative. For each country and each day, we followed Cori et al. and expressed the total infectiousness of the infected individuals as the weighted sum of the past incident cases [18], where the weight parameters were derived from the cumulative distribution function of COVID-19’s serial interval based on Nishiura et al.’s estimate [19]. We assumed the offspring distribution to follow a negative binomial distribution with mean *μ* = 2 in the absence of stay-at-home order, and mean *μ*^′^ = 1.0 or 1.5 once the stay-at-home order came into force, where we created two scenarios for the value of *μ*′. The over-dispersion parameter (denoted by *k*) of the offspring distribution was assumed to be time-invariant, and we tested the sensitivity of our simulation results with respect to the estimated value of *k* obtained from previous studies, namely, 0.10 by Endo et al [20], and 0.58 by Bi et al [21]. Hence, there are a total of four combinations of parameter values of *μ*′ and *k*, and under each combination, we ran the simulation algorithm following Churcher et al. [22] and derived the estimated probability of reaching 10,000 cases by the end of March, April, May, and June respectively for each country. (Refer to Figure 1 for the schematic overview of the methods, the Additional file 2 for the implementation details, and the Additional file 4 for the R code)

## Results

**Fig. 2:**
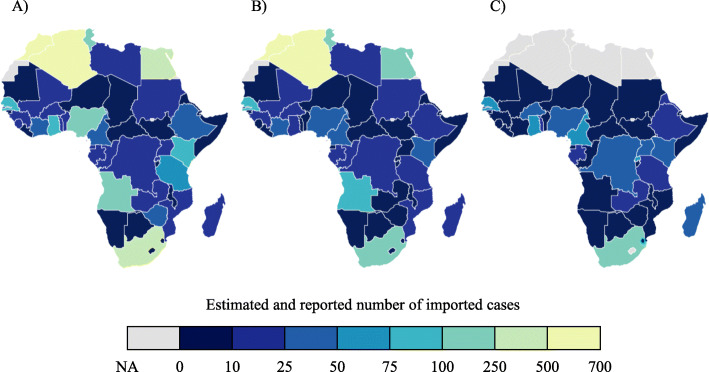
Estimated and reported number of imported COVID-19 cases. The first two subplots show the posterior median estimates of the number of COVID-19 cases imported from (**a**) all the 12 major epicentres in Europe and America, and (**b**) 10 epicentres only, after excluding United Kingdom and United States to create a more conservative estimate (Refer to Methods for more details). Note that in our estimates we only considered imported cases who had acquired infections prior to the travel ban coming into force. Subplot (**c**) shows the number of imported cases in each sub-Saharan African country that were reported by 31^st^ March 2020 based on data collated by Skrip et al. (Countries not included in Skrip et al. were coloured in grey).

We estimated Morocco, Algeria, South Africa, Egypt, Tunisia, and Nigeria as having the largest number of COVID-19 cases imported from the 12 new epicentres in Europe and America (Table 1 and Figure 2). All of these countries had their lower bound estimate of the imported case count exceeding 100 (Table 1). By contrast, nine countries (e.g. Lesotho, Eswatini, and South Sudan) were found to have a very low risk of importation, with the upper bound estimate of the imported case count below 10 (Table 1). For some countries such as Angola, the reported number of imported cases was substantially lower than our posterior median estimate (Figure 2). In a more conservative scenario where United Kingdom and United States were excluded from the list of epicentre countries, the estimated number of imported cases did not change drastically for most countries, albeit with some exceptions such as Kenya, whose estimate decreased from 97 (95% CI: 75–120) to 27 (95% CI: 16–41) (Table 1). The data file for Table 1 has been included within Additional file 3.

**Table 1:** Estimated number of COVID-19 cases (with 95% credible interval) imported from the 12 new epicentres in Europe and America (second column), and after excluding United Kingdom and United States from the list of epicentre countries (third column) to create a more conservative estimate (refer to Methods for more details). Note that in our estimates we only considered imported cases who had acquired infections prior to the travel ban coming into force

Country	Estimated imported case count from 12 epicentres	Estimated imported case count from 10 epicentres
Algeria	671 (489–891)	630 (449–851)
Angola	110 (48–227)	95 (34–212)
Benin	12 (6–20)	10 (4–18)
Botswana	4 (1–9)	1 (0–4)
Burkina Faso	15 (7–24)	13 (6–22)
Burundi	2 (0–7)	2 (0–5)
Cabo Verde	116 (83–173)	55 (27–109)
Cameroon	38 (25–54)	29 (18–44)
Central African Republic	3 (0–7)	3 (0–7)
Chad	3 (0–8)	2 (0–6)
Comoros	2 (0–6)	2 (0–6)
Congo	18 (9–29)	14 (6–25)
Congo DRC	22 (12–36)	17 (8–30)
Côte d'Ivoire	47 (29–68)	39 (22–60)
Djibouti	7 (2–13)	5 (1–10)
Egypt	287 (233–353)	173 (125–231)
Equatorial Guinea	12 (5–22)	9 (3–18)
Eritrea	3 (0–8)	1 (0–4)
Eswatini	0 (0–2)	0 (0–1)
Ethiopia	45 (32–61)	20 (10–31)
Gabon	16 (7–26)	14 (6–24)
Gambia	24 (14–35)	7 (2–14)
Ghana	77 (58–98)	16 (8–26)
Guinea	16 (8–25)	13 (6–22)
Guinea-Bissau	10 (3–24)	10 (2–24)
Kenya	97 (75–120)	27 (16–41)
Lesotho	0 (0–2)	0 (0–1)
Liberia	6 (2–12)	2 (0–6)
Libya	14 (4–36)	13 (3–34)
Madagascar	21 (11–34)	19 (10–31)
Malawi	7 (2–13)	1 (0–4)
Mali	23 (13–36)	21 (11–33)
Mauritania	6 (2–12)	5 (1–11)
Mauritius	122 (94–154)	66 (44–93)
Mayotte	6 (2–13)	6 (2–13)
Morocco	742 (575–959)	555 (391–765)
Mozambique	24 (11–46)	19 (7–40)
Namibia	10 (4–17)	6 (2–12)
Niger	8 (3–14)	6 (2–13)
Nigeria	160 (130–192)	28 (17–41)
Rwanda	12 (6–20)	5 (1–11)
Réunion	75 (46–114)	74 (45–113)
Sao Tome and Principe	8 (1–21)	8 (1–20)
Senegal	96 (69–129)	83 (57–115)
Seychelles	35 (23–49)	22 (12–34)
Sierra Leone	14 (7–22)	2 (0–6)
Somalia	9 (4–16)	2 (0–6)
South Africa	342 (287–404)	119 (89–159)
South Sudan	2 (0–6)	1 (0–3)
Sudan	23 (13–37)	12 (5–24)
Tanzania	58 (42–75)	23 (13–36)
Togo	11 (5–19)	8 (3–15)
Tunisia	214 (157–285)	195 (137–264)
Uganda	16 (8–25)	7 (2–13)
Zambia	15 (8–24)	3 (0–7)
Zimbabwe	30 (20–42)	3 (0–7)

**Fig. 3:**
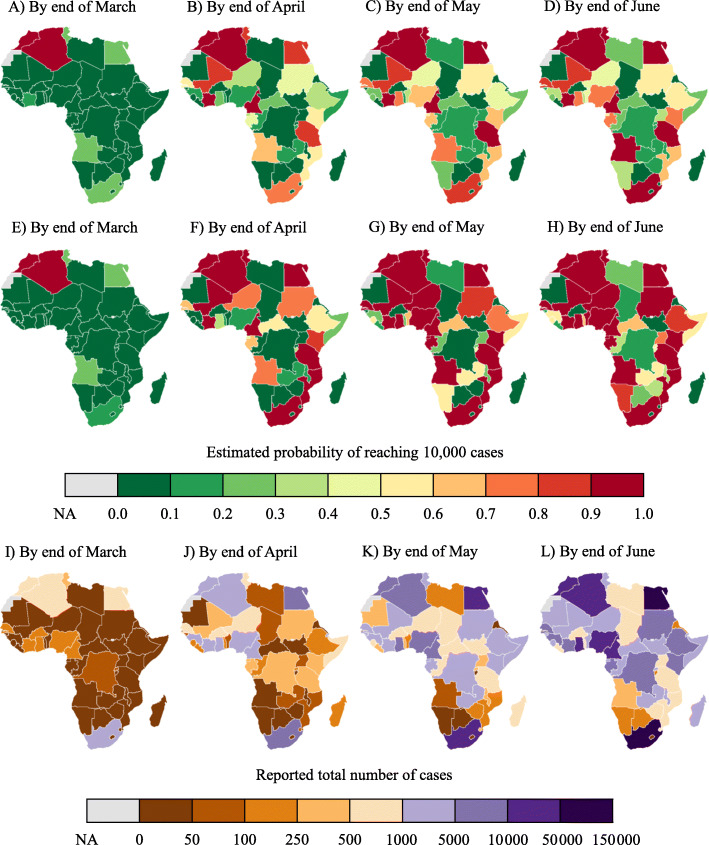
Estimated probability of reaching 10,000 cases as well as the reported total number of cases by each country (Stay-at-home order was assumed to reduce the reproduction number to 1.0). Reproduction number in the absence of stay-at-home order in each country was assumed to be 2. The over-dispersion parameter of the offspring distribution was (**a**–**d**) 0.10 and (**e**–**h**) 0.58 respectively. Reported total number of cases (**i**–**l**) were extracted from the World Health Organization’s situation reports.

**Fig. 4:**
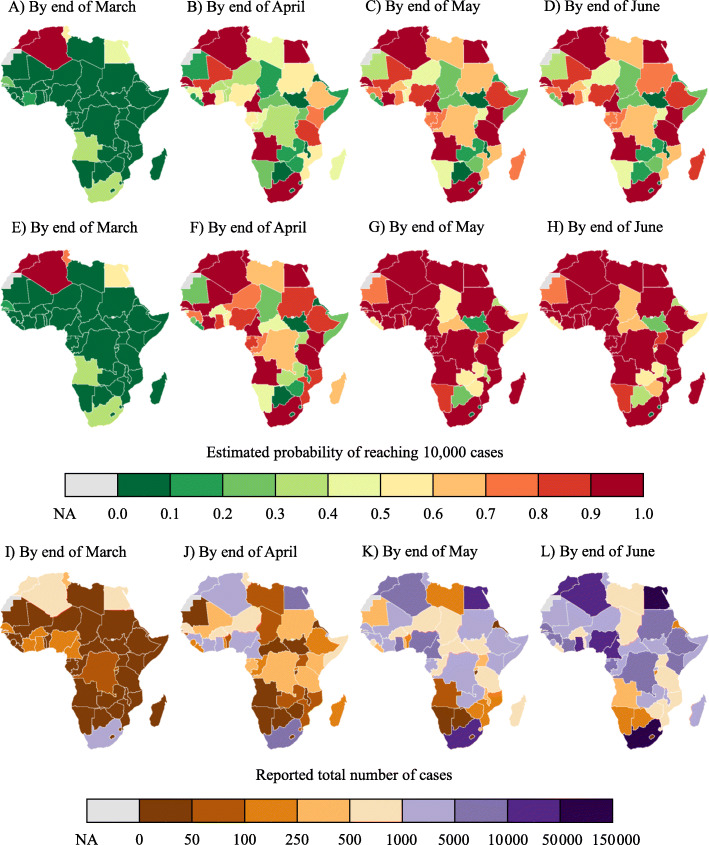
Estimated probability of reaching 10,000 cases as well as the reported total number of cases by each country (Stay-at-home order was assumed to reduce the reproduction number to 1.5). Reproduction number in the absence of stay-at-home order in each country was assumed to be 2. The over-dispersion parameter of the offspring distribution was (**a**–**d**) 0.10 and (**e**–**h**) 0.58 respectively. Reported total number of cases (**i**–**l**) were extracted from the World Health Organization’s situation reports.

Based on our 1,000 simulations of the onward SARS-CoV-2 spread, both Morocco and Algeria’s estimated probability of reaching 10,000 cases by end of March was close to 100% under all the four scenarios that we considered, whilst the reported total number of cases in each country by end of March was ~500 (Figures 3 and 4). We found the numbers of countries with a higher-than-50% estimated probability of reaching 10,000 cases by end of March, April, May, and June to be 2, 13, 22, and 24 respectively (Figure 3) under the assumption that reproduction number is reduced to 1.0 by stay-at-home order and the offspring distribution is highly over-dispersed (i.e. *k* = 0.10). This scenario is considered to be the most conservative in terms of the estimated risk of reaching 10,000 cases in general (Figures 3 and 4), and yet the total numbers of countries that had reported over 10,000 cases by end of March, April, May, and June were only 0, 0, 2, and 7 respectively. Moreover, six countries (Angola, Gambia, Mauritius, Mozambique, Sao Tome and Principe, and Tanzania) were found to have reported less than 1,000 cases by end of June whilst the estimated probability of reaching 10,000 cases by then was higher than 50% even under the most optimistic scenario (Figure 3), suggesting that a very substantial number of cases may have been undetected. It should be noted that when the over-dispersion parameter *k* was changed from 0.10 to 0.58 there was a significant increase in our risk estimates in the later months (i.e. May and June) (Figures 3 and 4), and the 95% credible interval for the date at which 10,000 cases are reached in each country was also narrower in general under a less over-dispersed offspring distribution (i.e. *k* = 0.58) (Tables 2 and 3). Nonetheless, the ranking of the estimated risk of reaching 10,000 cases in each country was largely consistent across different combinations of model parameter values (Figures 3 and 4).

**Table 2:** Summary statistics for the estimated date by which 10,000 cases are reached in each African country. Reproduction numbers used for the simulation were 2.0 before and 1.0 after stay-at-home order came into force in each country. Estimates were derived under two scenarios, where the over-dispersion parameter of the offspring distribution was set at 0.10 (higher over-dispersion) and 0.58 (lower over-dispersion) respectively. Simulations were performed until 30^th^ June, or 10,000 cases are reached, whichever is earlier, based on 1,000 model runs

Country	Over-dispersion parameter = 0.10					Over-dispersion parameter = 0.58				
	2.5 Percentile	25 Percentile	50 Percentile	75 Percentile	97.5 Percentile	2.5 Percentile	25 Percentile	50 Percentile	75 Percentile	97.5 Percentile
Algeria	03-17	03-19	03-21	03-22	03-27	03-18	03-20	03-20	03-21	03-24
Angola	03-21	03-31	04-16	05-29	>06-30	03-24	03-30	04-08	04-24	06-06
Benin	04-05	05-26	>06-30	>06-30	>06-30	04-18	05-22	05-28	06-05	>06-30
Botswana	05-05	>06-30	>06-30	>06-30	>06-30	05-26	06-22	>06-30	>06-30	>06-30
Burkina Faso	05-20	>06-30	>06-30	>06-30	>06-30	>06-30	>06-30	>06-30	>06-30	>06-30
Burundi	04-11	>06-30	>06-30	>06-30	>06-30	04-12	04-26	>06-30	>06-30	>06-30
Cabo Verde	03-24	04-10	05-11	>06-30	>06-30	03-28	04-10	04-25	05-25	>06-30
Cameroon	03-30	04-06	04-12	04-19	>06-30	03-31	04-06	04-09	04-12	04-19
Central African Republic	04-07	05-10	>06-30	>06-30	>06-30	04-09	04-20	04-30	>06-30	>06-30
Chad	04-14	>06-30	>06-30	>06-30	>06-30	04-23	>06-30	>06-30	>06-30	>06-30
Comoros	04-07	>06-30	>06-30	>06-30	>06-30	04-09	04-23	05-16	>06-30	>06-30
Congo	04-03	>06-30	>06-30	>06-30	>06-30	04-14	06-09	>06-30	>06-30	>06-30
Congo DRC	04-13	>06-30	>06-30	>06-30	>06-30	05-07	>06-30	>06-30	>06-30	>06-30
Côte d'Ivoire	03-27	04-03	04-08	04-13	>06-30	03-30	04-03	04-06	04-08	04-16
Djibouti	05-26	>06-30	>06-30	>06-30	>06-30	05-30	06-12	06-27	>06-30	>06-30
Egypt	03-23	03-31	04-07	04-19	06-16	03-26	04-01	04-05	04-11	05-01
Equatorial Guinea	04-04	04-27	05-13	>06-30	>06-30	04-11	04-26	05-01	05-08	>06-30
Eritrea	04-24	>06-30	>06-30	>06-30	>06-30	05-19	>06-30	>06-30	>06-30	>06-30
Eswatini	>06-30	>06-30	>06-30	>06-30	>06-30	>06-30	>06-30	>06-30	>06-30	>06-30
Ethiopia	04-02	04-20	06-08	>06-30	>06-30	04-04	04-14	04-27	06-01	>06-30
Gabon	03-30	04-12	05-16	06-19	>06-30	04-03	04-10	04-21	05-14	06-04
Gambia	04-04	04-18	05-10	>06-30	>06-30	04-08	04-15	04-20	04-28	>06-30
Ghana	04-02	04-29	05-08	>06-30	>06-30	04-09	04-27	05-03	05-08	05-22
Guinea	04-02	06-20	>06-30	>06-30	>06-30	04-13	06-10	06-29	>06-30	>06-30
Guinea-Bissau	04-08	>06-30	>06-30	>06-30	>06-30	04-16	>06-30	>06-30	>06-30	>06-30
Kenya	03-29	04-08	04-21	06-28	>06-30	03-31	04-07	04-13	04-24	>06-30
Lesotho	>06-30	>06-30	>06-30	>06-30	>06-30	>06-30	>06-30	>06-30	>06-30	>06-30
Liberia	04-16	>06-30	>06-30	>06-30	>06-30	04-19	>06-30	>06-30	>06-30	>06-30
Libya	04-16	>06-30	>06-30	>06-30	>06-30	04-29	>06-30	>06-30	>06-30	>06-30
Madagascar	04-28	>06-30	>06-30	>06-30	>06-30	06-19	>06-30	>06-30	>06-30	>06-30
Malawi	04-12	>06-30	>06-30	>06-30	>06-30	04-14	05-26	>06-30	>06-30	>06-30
Mali	03-30	04-08	04-14	04-24	>06-30	04-01	04-08	04-11	04-15	04-24
Mauritania	04-24	>06-30	>06-30	>06-30	>06-30	05-08	>06-30	>06-30	>06-30	>06-30
Mauritius	03-28	04-26	06-01	06-15	>06-30	04-08	04-29	05-18	06-04	06-16
Mayotte	05-26	>06-30	>06-30	>06-30	>06-30	>06-30	>06-30	>06-30	>06-30	>06-30
Morocco	03-16	03-18	03-20	03-22	03-29	03-17	03-19	03-19	03-21	03-25
Mozambique	03-29	04-11	04-24	>06-30	>06-30	04-01	04-08	04-15	04-21	>06-30
Namibia	04-19	05-27	>06-30	>06-30	>06-30	05-04	05-22	05-30	06-11	>06-30
Niger	04-05	04-22	>06-30	>06-30	>06-30	04-07	04-16	04-23	05-01	>06-30
Nigeria	04-03	05-12	05-22	06-11	>06-30	04-10	05-11	05-16	05-21	06-02
Rwanda	05-17	>06-30	>06-30	>06-30	>06-30	05-21	06-01	06-15	>06-30	>06-30
Réunion	03-29	04-24	05-19	05-28	>06-30	04-06	04-25	05-13	05-20	05-28
Sao Tome and Principe	03-31	04-18	05-02	>06-30	>06-30	04-03	04-14	04-21	04-30	>06-30
Senegal	03-25	04-10	04-27	06-06	>06-30	03-31	04-11	04-19	05-04	>06-30
Seychelles	>06-30	>06-30	>06-30	>06-30	>06-30	>06-30	>06-30	>06-30	>06-30	>06-30
Sierra Leone	04-12	>06-30	>06-30	>06-30	>06-30	04-26	05-13	05-28	>06-30	>06-30
Somalia	04-15	>06-30	>06-30	>06-30	>06-30	04-18	04-30	05-19	>06-30	>06-30
South Africa	03-24	04-02	04-13	05-03	06-18	03-27	04-02	04-07	04-14	05-06
South Sudan	>06-30	>06-30	>06-30	>06-30	>06-30	>06-30	>06-30	>06-30	>06-30	>06-30
Sudan	04-05	04-16	05-10	>06-30	>06-30	04-08	04-15	04-19	05-01	>06-30
Tanzania	03-30	04-09	04-15	04-24	>06-30	04-03	04-08	04-11	04-15	04-24
Togo	04-05	06-23	>06-30	>06-30	>06-30	04-11	05-31	06-25	>06-30	>06-30
Tunisia	03-21	03-31	04-08	04-22	05-27	03-25	03-31	04-05	04-12	05-02
Uganda	04-09	06-05	>06-30	>06-30	>06-30	04-26	06-01	06-09	>06-30	>06-30
Zambia	04-09	>06-30	>06-30	>06-30	>06-30	04-12	05-12	05-31	>06-30	>06-30
Zimbabwe	05-03	>06-30	>06-30	>06-30	>06-30	06-10	06-28	>06-30	>06-30	>06-30

**Table 3:** Summary statistics for the estimated date by which 10,000 cases are reached in each African country. Reproduction numbers used for the simulation were 2.0 before and 1.5 after stay-at-home order came into force in each country. Estimates were derived under two scenarios, where the over-dispersion parameter of the offspring distribution was set at 0.10 (higher over-dispersion) and 0.58 (lower over-dispersion) respectively. Simulations were performed until 30^th^ June, or 10,000 cases are reached, whichever is earlier, based on 1,000 model runs

Country	Over-dispersion parameter = 0.10					Over-dispersion parameter = 0.58				
	2.5 Percentile	25 Percentile	50 Percentile	75 Percentile	97.5 Percentile	2.5 Percentile	25 Percentile	50 Percentile	75 Percentile	97.5 Percentile
Algeria	03-17	03-19	03-21	03-22	03-25	03-18	03-20	03-20	03-21	03-24
Angola	03-22	03-29	04-05	04-12	05-06	03-25	03-30	04-03	04-08	04-20
Benin	04-04	04-25	05-20	>06-30	>06-30	04-07	04-19	04-28	05-09	>06-30
Botswana	04-18	>06-30	>06-30	>06-30	>06-30	04-21	05-22	>06-30	>06-30	>06-30
Burkina Faso	04-04	04-25	05-14	>06-30	>06-30	04-12	04-24	05-01	05-10	06-16
Burundi	04-10	>06-30	>06-30	>06-30	>06-30	04-13	04-26	>06-30	>06-30	>06-30
Cabo Verde	03-24	04-03	04-10	04-19	05-30	03-27	04-03	04-07	04-12	04-25
Cameroon	03-29	04-06	04-11	04-18	>06-30	03-31	04-05	04-08	04-11	04-19
Central African Republic	04-07	05-07	>06-30	>06-30	>06-30	04-08	04-21	05-02	>06-30	>06-30
Chad	04-10	>06-30	>06-30	>06-30	>06-30	04-13	05-02	05-24	>06-30	>06-30
Comoros	04-09	>06-30	>06-30	>06-30	>06-30	04-09	04-22	05-09	>06-30	>06-30
Congo	04-01	04-18	05-02	06-10	>06-30	04-06	04-16	04-22	05-01	05-30
Congo DRC	04-03	04-22	05-08	>06-30	>06-30	04-07	04-18	04-25	05-05	>06-30
Côte d'Ivoire	03-27	04-04	04-08	04-14	05-14	03-30	04-03	04-06	04-08	04-14
Djibouti	04-13	05-16	>06-30	>06-30	>06-30	04-16	05-06	05-18	06-03	>06-30
Egypt	03-23	03-28	04-01	04-05	04-15	03-25	03-29	03-31	04-02	04-06
Equatorial Guinea	04-03	04-19	05-01	>06-30	>06-30	04-09	04-18	04-23	04-30	>06-30
Eritrea	04-19	>06-30	>06-30	>06-30	>06-30	04-18	05-19	>06-30	>06-30	>06-30
Eswatini	>06-30	>06-30	>06-30	>06-30	>06-30	05-22	>06-30	>06-30	>06-30	>06-30
Ethiopia	03-31	04-13	04-24	05-11	>06-30	04-04	04-11	04-17	04-23	05-14
Gabon	03-31	04-12	04-24	05-24	>06-30	04-02	04-09	04-14	04-22	05-17
Gambia	04-05	04-17	05-15	>06-30	>06-30	04-08	04-15	04-21	04-28	>06-30
Ghana	04-01	04-18	04-28	06-03	>06-30	04-05	04-15	04-21	04-26	05-10
Guinea	04-02	04-20	05-07	>06-30	>06-30	04-06	04-17	04-24	05-02	05-29
Guinea-Bissau	04-01	04-28	05-26	>06-30	>06-30	04-05	04-22	05-03	05-17	>06-30
Kenya	03-29	04-07	04-15	04-29	>06-30	04-01	04-07	04-11	04-16	05-01
Lesotho	>06-30	>06-30	>06-30	>06-30	>06-30	05-24	>06-30	>06-30	>06-30	>06-30
Liberia	04-12	>06-30	>06-30	>06-30	>06-30	04-14	05-05	05-29	>06-30	>06-30
Libya	04-06	04-22	05-07	>06-30	>06-30	04-11	04-21	04-27	05-04	>06-30
Madagascar	04-03	04-21	05-05	05-29	>06-30	04-08	04-19	04-26	05-03	05-24
Malawi	04-13	>06-30	>06-30	>06-30	>06-30	04-15	05-12	>06-30	>06-30	>06-30
Mali	03-30	04-08	04-15	04-25	>06-30	04-01	04-08	04-11	04-15	04-25
Mauritania	04-09	05-09	>06-30	>06-30	>06-30	04-12	04-29	05-12	06-11	>06-30
Mauritius	03-27	04-05	04-11	04-18	05-09	03-31	04-05	04-08	04-12	04-21
Mayotte	04-07	05-06	>06-30	>06-30	>06-30	04-14	04-29	05-11	05-26	>06-30
Morocco	03-16	03-18	03-20	03-21	03-25	03-17	03-19	03-20	03-21	03-23
Mozambique	03-28	04-11	04-23	>06-30	>06-30	04-01	04-09	04-14	04-21	>06-30
Namibia	04-06	05-03	>06-30	>06-30	>06-30	04-09	04-23	05-04	05-14	>06-30
Niger	04-04	04-21	>06-30	>06-30	>06-30	04-08	04-17	04-23	05-02	>06-30
Nigeria	03-31	04-14	04-25	05-10	>06-30	04-05	04-13	04-19	04-25	05-08
Rwanda	04-15	05-13	>06-30	>06-30	>06-30	04-21	05-06	05-14	05-27	>06-30
Réunion	03-26	04-04	04-09	04-16	05-08	03-30	04-04	04-07	04-11	04-20
Sao Tome and Principe	04-02	04-18	05-06	>06-30	>06-30	04-03	04-14	04-21	04-30	>06-30
Senegal	03-25	04-02	04-07	04-13	04-28	03-28	04-02	04-05	04-08	04-15
Seychelles	>06-30	>06-30	>06-30	>06-30	>06-30	>06-30	>06-30	>06-30	>06-30	>06-30
Sierra Leone	04-11	>06-30	>06-30	>06-30	>06-30	04-13	05-02	05-17	>06-30	>06-30
Somalia	04-13	>06-30	>06-30	>06-30	>06-30	04-16	04-30	05-17	>06-30	>06-30
South Africa	03-24	03-30	04-03	04-08	04-20	03-26	03-30	04-02	04-04	04-10
South Sudan	04-29	>06-30	>06-30	>06-30	>06-30	04-30	06-15	>06-30	>06-30	>06-30
Sudan	04-05	04-17	04-29	>06-30	>06-30	04-08	04-15	04-20	04-26	05-28
Tanzania	03-29	04-08	04-14	04-23	>06-30	04-02	04-08	04-11	04-15	04-24
Togo	04-03	04-26	06-12	>06-30	>06-30	04-08	04-20	04-29	05-11	>06-30
Tunisia	03-21	03-27	03-30	04-03	04-13	03-24	03-27	03-29	03-31	04-05
Uganda	04-05	04-28	>06-30	>06-30	>06-30	04-12	04-26	05-05	05-21	>06-30
Zambia	04-09	>06-30	>06-30	>06-30	>06-30	04-10	04-26	05-21	>06-30	>06-30
Zimbabwe	04-15	>06-30	>06-30	>06-30	>06-30	04-16	05-07	05-24	>06-30	>06-30

## Discussion

Our study has estimated the size of the second wave of COVID-19 importations in each African country from the 12 major epicentres in Europe and America. This allows us to narrow the knowledge gap between the observed and actual number of importations, so that the unfolding of the epidemic seeded by the imported cases can be better projected especially in countries with very low testing capacities.

In the first wave of importations of cases from Wuhan, China, to other places outside China we estimated that most places at risk were in Asia, Europe and USA [23]. Though there were links between China and African countries, these were fewer than those between China and the rest of Asia, Europe and USA [23]. The shut down in China severely curtailed continuing importations out of China and so these importations rapidly stopped.

Lower initial importations into Africa compared to Asia and Europe certainly tallies with what has been seen. There have been very few reported cases in Africa in the first wave of importations, and no reports of onward transmission. There was much discussion at the time whether the lack of reported imported cases in Africa was because imported cases were not being picked up. This may be some of the story, but our analysis would suggest that this was not the whole story, and it was more that the early risk of importation into Africa was lower than other places [23]. However the results we present in this paper estimate that this risk has dramatically increased with the spread of the virus in Europe and the USA. This also tallies with what we have seen, as countries in Africa started to report their first imported cases from Europe and the USA [4]. As of July 15^th^ 2020, South Africa had reported the highest number of cases at 298,292 [4], and we estimated South Africa to have had one of the highest numbers of imported cases from the new epi-centres, although it was also rated highest at risk in Africa of importations from China in previous analysis [6]. Senegal is one of the countries for whom the risk has notably increased from the risk of importation from China as estimated in previous analyses [6, 23]. We only considered importations from the major epicentres in Europe and America, and so the number of importations from all countries will be even higher. Hence, countries whose reported total number of imported cases was substantially lower than our estimates were likely to have severely under-detected COVID-19 cases imported from the new epi-centres. However it could also be that the difference between the reported and estimated numbers was due to missing travel history information among the reported cases.

Our study provides countries with information on the estimated timing of reaching 10,000 cases, which complements previous work by Pearson et al. [8] Our analyses further accounted for the estimated number and timing of imported cases, both being insensitive to the case reporting rates that vary across different countries. We also incorporated the effects of quarantine and stay-at-home order on the onward transmission, as well as the impact of different input parameter values on the simulation results. Notably, we found that a highly over-dispersed offspring distribution would lead to a relatively lower estimated magnitude of onward transmission, as well as higher uncertainty in the timing of reaching 10,000 cases. The former can be explained by a larger probability of producing zero offspring per COVID-19 case albeit a higher occurrence of super-spreading events. Whilst it is challenging to produce accurate estimates of the over-dispersion parameter and reproduction number in each country, the different scenarios created in our sensitivity analysis overall reflect our current understanding of the possible range of parameter values based on the data available [20, 21, 24]. Importantly, our results highlight countries whose reported numbers of cases remained substantially lower than the model estimates under all the scenarios that we considered, including some (e.g. Tanzania) where stringent measures such as stay-at-home order have yet to be implemented at the time of writing.

Many countries in Africa have considerable experience in dealing with other infectious disease outbreaks, most notably Ebola, and will be able to call upon that experience for COVID-19. Countries hit in this third wave of transmission, including those in Africa have some advantage as there have been a variety of responses from around the world from which to assess what to do or not to do. However there will need to be consideration of how effective measures can be adapted to different settings [25]. Issues such as high HIV prevalence in some countries, and a younger demographic may both affect the cases and deaths observed in different ways. Recent work has yielded important insights into the age-dependent susceptibility to infection as well as symptomatic rates [26], and more studies are urgently needed from different locations to parameterise this in models for different countries and to inform how best to respond in each local context.

Many countries in Africa are on high alert for incoming cases from Europe and USA, taking measures such as quarantine of arrivals or shutting down travel from affected countries. However as travel is either maintained or reopened between countries closer by, risk of importations from other countries should continue to be considered. Close attention should therefore be paid to where will be the next epicentre, perhaps within Africa, and how this could translate into imported cases for each country, particularly for those countries that we estimate to have experienced lower numbers of imported cases previously and therefore lower onward transmission.

Not accounted for in our study currently is the impact of less stringent interventions on the local SARS-CoV-2 spread, such as the effect of prohibiting large public gatherings, closure of social venues and schools, and restrictions on inter-district travels. It is still unclear as to whether and to what extent these interventions were effective in their local context, and hence in our simulations we only considered stay-at-home order for all non-essential workers as an effective intervention to reduce local transmission. Future modelling work considering the impact of different interventions in different places will be vital for determining how each country can continue to respond.

In addition, we have made simplifying assumptions about the change in travel patterns in response to the pandemic in each African country relative to that in Singapore, due to the unavailability of 2020 flight data. We were also unable to account for the complex effects of population structure on the onward transmission of the virus in each country. Despite these limitations, most of our model assumptions throughout the analyses have been fairly conservative to avoid inflating the projections of the SARS-CoV-2 spread. For example, the reported number of imported cases in Singapore was assumed to be complete, and the risk of returning citizens carrying SARS-CoV-2 after travel restrictions came into force in each African country was also not included. Simulations of the onward spread of the virus were based on the estimated number of imported cases from the selected 10 epicentre countries, and stay-at-home order was assumed to be effective (reproduction numbers being 1.5 and 1.0 in the two scenarios we considered). In light of these conservative assumptions, any countries found to have a high probability of reaching 10,000 cases by end May or June under the most optimistic scenario—especially those with very limited cases detected—need urgent actions.

## Conclusions

In conclusion, our study provides model estimates of the number of COVID-19 cases imported from major epicentres in Europe and America to each country in Africa, as well as simulation results of the onward epidemic spread. Our results highlight particular countries that are likely to reach (or have reached) 10,000 cases far earlier than the reported data suggest, calling for the prioritization of resources to mitigate the further spread of the epidemic.

## Supplementary information

**Additional file 1.** Start dates of travel/internal restrictions in each African country.

**Additional file 2.** Supporting information.

**Additional file 3.** Data file for Table 1.

**Additional file 4.** R code.

## Data Availability

Data on the reported number of imported cases in Singapore are available from https://www.moh.gov.sg/covid-19/past-updates Flight data used in this study were purchased from the Official Airline Guide. Government response data used in this study have been included within the Additional file 1. Supporting information has been included within the Additional file 2. Data file for Table 1 has been included within Additional file 3. R code has been included within Additional file 4.

## Abbreviations

COVID-19: Coronavirus disease 2019
SARS-CoV-2: Severe acute respiratory syndrome coronavirus 2
