# Supplementary material for: Importations of COVID-19 into African countries and risk of onward spread
Source: BMC Infect Dis. 2020 Aug 13;20:598. doi: 10.1186/s12879-020-05323-w (PMC7424562; doi:10.1186/s12879-020-05323-w)
Supplement: Supplementary file 2 — Additional file 2. Supporting information. [file 12879_2020_5323_MOESM2_ESM.docx]

Importations of COVID-19 into African countries and risk of onward spread

Haoyang Sun ^1, §^, Borame L Dickens ^1^, Alex R Cook ^1^, Hannah E Clapham ^1, §^

^1^ Saw Swee Hock School of Public Health, National University of Singapore, 12 Science Drive 2, Singapore 117549, Republic of Singapore

^§^ Correspondence to:

Haoyang Sun. Saw Swee Hock School of Public Health, National University of Singapore, 12 Science Drive 2, Singapore 117549, Republic of Singapore. Email: [haoyang.sun@nus.edu.sg](mailto:haoyang.sun@nus.edu.sg).

Hannah E Clapham. Saw Swee Hock School of Public Health, National University of Singapore, 12 Science Drive 2, Singapore 117549, Republic of Singapore. Email: [hannah.clapham@nus.edu.sg](mailto:hannah.clapham@nus.edu.sg).

Supporting Information

Statistical analyses

*Estimating the number of imported cases*

For each African country$r$, we denote the daily number of air passengers that arrived from an epicentre country$e$ by$v_{e\to r}^{(t)} (t=t_{e}, t_{e}+1, \ldots, T_{e\to r})$, where$t_{e}$ refers to the start date of the COVID-19 epidemic in the epicentre country$e$, and$T_{e\to r}$ refers to the last day that non-citizens and non-residents travelling from country$e$ were allowed to enter country$r$. Each day the probability that an air passenger travelling from country$e$ to country$r$ was an imported case is denoted by$p_{e}^{(t)}$, which we assume to be dependent on both the origin country$e$ and time$t$, but independent from the destination country$r$. In other words, the destination location was assumed to have a negligible impact on the risk of a traveller being an imported case, controlling for the origin location and travel date. Hence, the total number of COVID-19 cases imported from an epicentre country$e$ to an African country$r$ by the time the travel ban came into force (denoted by$M_{e\to r}$ below) can be expressed as the sum of the following independent binomial random variables (i.e. daily number of cases imported from$e$ to$r$):

$$M_{e\to r}=\sum_{t=t_{e}}^{T_{e\to r}} M_{e\to r}^{\left( t \right)},$$

$$M_{e\to r}^{\left( t \right)}\sim Bin\left( v_{e\to r,}^{\left( t \right)} p_{e}^{\left( t \right)} \right).$$

Given that in most cases the daily number of air travellers$v_{e\to r}^{\left( t \right)}$ before the travel ban came into force was reasonably large (≥20), the random variable$M_{e\to r}$ can be approximated using a Poisson distribution:

$$M_{e\to r} \dot{\sim} Po\left( \sum_{t=t_{e}}^{T_{e\to r}} v_{e\to r}^{\left( t \right)}\cdot p_{e}^{\left( t \right)} \right).$$

We used the imported COVID-19 case data reported by Singapore as well as flight data to provide a conservative estimate for$M_{e\to r}$, under the assumption that Singapore, being one of the countries with the highest surveillance capacity^1^, has detected all the imported cases. Owing to the delay from infection to hospital admission, we considered all cases imported from country$e$ to Singapore that were *reported* by date$(T_{e\to r}+9)$ (hereinafter denoted as${SG}_{e,r}$) based on Linton et al.’s estimated mean incubation period and time from illness onset to hospital admission^2^. We assumed that the ratio between the daily number of air travellers from epicentre$e$ to country$r$ and to Singapore remained stable in the presence of the changes in flight pattern in response to the COVID-19 pandemic (i.e. the percentage change in air passenger volumes was consistent across the two country pairs). Hence, the ratio between the expected numbers of cases imported from epicentre$e$ to African country$r$ and to Singapore can be derived using the March 2017 flight data:

$$\frac{\mathbb{E[}M_{e\to r}]}{\mathbb{E[}{SG}_{e,r}]}= \frac{\sum_{t=t_{e}}^{T_{e\to r}} v_{e\to r}^{\left( t \right)}\cdot p_{e}^{\left( t \right)}}{\sum_{t=t_{e}}^{T_{e\to r}} v_{e\to SG}^{\left( t \right)}\cdot p_{e}^{\left( t \right)}}=\frac{\sum_{t in Mar 17} v_{e\to r}^{\left( t \right)}}{\sum_{t in Mar 17} v_{e\to SG}^{\left( t \right)}}.$$

This allows us to model$M_{e\to r}$ (and${SG}_{e,r}$) as Poisson random variables with mean parameters proportional to the numbers of air passengers travelling from epicentre$e$ to country$r$ (and to Singapore) using the March 2017 flight data (i.e. $\mathbb{E}\left[ M_{e\to r} \right]$ now becomes a function of the sum of$v_{e\to r}^{\left( t \right)}$ over March 2017, and similarly for$\mathbb{E[}{SG}_{e,r}]$):

$$M_{e\to r}\dot{\sim} Po\left( \beta_{e,r}\cdot\sum_{t in Mar 17} v_{e\to r}^{\left( t \right)} \right),$$

$${SG}_{e,r}\dot{\sim} Po\left( \beta_{e,r}\cdot\sum_{t in Mar 17} v_{e\to SG}^{\left( t \right)} \right).$$

Here, $\beta_{e,r}$ refers to the proportionality constant to be estimated using the reported value of${SG}_{e,r}$ and flight data, and was assigned a uniform prior with support (0, 1). We performed Markov Chain Monte Carlo to sample from the posterior distribution of$\beta_{e,r}$ using the JAGS software^3^. A total of 10 chains were run in parallel, each with 2,000 iterations burn-in and 15,000 iterations thinned and subsequently merged to obtain a posterior sample of size 5,000. The Geweke’s statistics for all parameters$\beta_{e,r}$’s ranged from -2.06 to 2.38^4^, and all the estimated Brooks & Gelman’s potential scale reduction factors were below 1.01^5^, both providing evidence for chain convergence. The posterior sample for all the model parameters was then used to estimate the uncertainty distribution of the total number of COVID-19 cases imported from the 12 major epicentres to each country.

In March 2020, a spike in the number of cases imported from United Kingdom and United States was observed in Singapore, which was partly due to the increase in the number of returning Singaporean students studying overseas^6^. This change in flight patterns, however, may not be applicable to all African countries. Therefore, to be even more conservative, we also derived the imported case count estimates excluding United Kingdom and United States from the 12 epicentre countries previously considered. The resulting estimates were subsequently used in the simulations of the onward spread of SARS-CoV-2 to get our estimates of case numbers over time.

*Simulating the onward transmission following importation*

We performed 1,000 simulations drawing from our estimated distribution of the number of imported cases to project the onward spread of SARS-CoV-2 in each country up to 30^th^ June 2020 or the date when we estimate 10,000 cases was reached, whichever was earlier. The time of infection for the cases imported from country$e$ to country$r$ was simulated via resampling from the reporting dates of the${SG}_{e,r}$ cases, which was then shifted backwards by 9 days to account for the delay from infection to hospital admission based on Linton et al.’s estimates^2^. To account for the effect of quarantine measures on the onward transmission, we only included the estimated imported cases who had acquired the infection prior to the mandatory quarantine of travellers coming into force, so that the estimation of local SARS-CoV-2 spread is conservative. Hence, following Cori et al.^7^, in each simulation$s$, the total infectiousness of the infected individuals in a country$r$ on day$t$ (denoted by$\tilde{I_{r}^{(t)}[s]}$) can be expressed as the weighted sum of the past incident cases (considered up to 14 days prior to$t$):

$$\tilde{I_{r}^{(t)}[s]}=\sum_{u=1}^{14} \omega_{u}\cdot I_{r}^{\left( t-u \right)}\left[ s \right].$$

In the equation above,$I_{r}^{\left( t-u \right)}\left[ s \right]$ denotes the number of incident cases in country$r$ on day$(t-u)$ generated in simulation$s$, which included both the number of incident local cases and the *effective* number of imported cases (i.e. after adjusted for the effect of mandatory quarantine measures, if applicable) whose *infection date* was$\left( t-u \right)$. The weight parameter$\omega_{u}$ can be derived from the cumulative distribution function of COVID-19’s serial interval based on Nishiura et al.’s estimate (denoted by$F(\cdot)$)^8^ (Refer to the section “Derivation of the weight parameters”):

$$\omega_{u}=\int_{u-1}^{u} F\left( \tau+1 \right)-F(\tau)d\tau.$$

We assumed the offspring distribution to follow a negative binomial distribution with mean $\mu=2$in the absence of stay-at-home order, and mean$\mu^{'}=1.0 \mathrm{or} 1.5$ once the stay-at-home order came into force, where we created two scenarios for the value of$\mu'$. The over-dispersion parameter (denoted by $k$) of the offspring distribution was assumed to be time-invariant, and we tested the sensitivity of our simulation results with respect to the estimated value of$k$ obtained from previous studies, namely, 0.10 by Endo et al^9^, and 0.58 by Bi et al^10^. Hence, there are a total of four combinations of parameter values of$\mu'$ and$k$, and under each combination, we followed the algorithm implemented by Churcher et al.^11^, where in each simulation$s$ we generated the number of incident local cases in country$r$ on day$t$ ($A_{r}^{(t)}\left[ s \right]$) as follows:

$$A_{r}^{(t)}\left[ s \right] \sim NB\left( \tilde{I_{r}^{\left( t \right)}\left[ s \right]}\cdot k, \left( 1+\frac{{R_{0}}_{r}^{\left( t \right)}}{k} \right)^{-1} \right).$$

The negative binomial distribution above modelled the number of failures before the$\tilde{I_{r}^{\left( t \right)}\left[ s \right]}\cdot k^{\mathrm{th}}$ success in a Bernoulli process with a success probability$\left( 1+\frac{{R_{0}}_{r}^{\left( t \right)}}{k} \right)^{-1}$ for each trial, where${R_{0}}_{r}^{\left( t \right)}$ denotes the instantaneous reproduction number in country$r$ on day$t$ as defined by Cori et al.^7^ and is equal to $\mu$ or$\mu'$ depending on the presence / absence of stay-at-home order as described earlier. Finally, based on the results obtained from the 1,000 simulations, we derived the estimated probability of reaching 10,000 cases by end of March, April, May, and June respectively for each African country.

**Derivation of the weight parameters**

Here we describe how the weight parameters$\omega_{u}'s$ can be derived from the cumulative distribution function of COVID-19’s serial interval based on Nishiura et al.’s estimate (denoted by$F(\cdot)$)^8^

Let$g\left( \tau\right):=F\left( \tau+1 \right)-F(\tau)$, and thus the weight parameter $\omega_{u}$ is given by the following equation (i.e. average of the$g(\cdot)$ values over all the time points within the interval$(u-1, u)$)

$$\omega_{u}=\lim_{n\to\infty} \frac{g\left( u-1+\frac{1}{n} \right)+g\left( u-1+\frac{2}{n} \right)+\ldots+g\left( u-1+\frac{n}{n} \right)}{n}.$$

And hence the above can be rewritten as:

$$\omega_{u}=\frac{1}{1}\int_{u-1}^{u} g\left( \tau\right)d\tau=\frac{1}{1}\int_{u-1}^{u} \left\{ F\left( \tau+1 \right)-F\left( \tau\right) \right\}d\tau.$$

The denominator in the equation above was 1 (day) since the weight parameter was derived for each day (instead of e.g. each week, and the serial interval was also expressed in days). The units of the left and right hand sides are now equal to each other (and the denominator 1 can be omitted for brevity).

References

1. Niehus R, Salazar PM De, Taylor A, Lipsitch M. Quantifying bias of COVID-19 prevalence and severity estimates in Wuhan, China that depend on reported cases in international travelers. medRxiv. 2020;

2. Linton NM, Kobayashi T, Yang Y, Hayashi K, Akhmetzhanov AR, Jung S, et al. Incubation Period and Other Epidemiological Characteristics of 2019 Novel Coronavirus Infections with Right Truncation: A Statistical Analysis of Publicly Available Case Data. J Clin Med [Internet]. 2020 Feb 17;9(2):538. Available from: https://www.mdpi.com/2077-0383/9/2/538

3. Plummer M. JAGS : A program for analysis of Bayesian graphical models using Gibbs sampling. In 2003. Available from: http://www.ci.tuwien.ac.at/Conferences/DSC-2003/

4. Geweke J. Evaluating the accuracy of sampling-based approaches to calculating posterior moments. In: Bayesian Statistics 4. Oxford, UK: Clarendon Press; 1992. p. 169–193.

5. Brooks SP, Gelman A. General methods for monitoring convergence of iterative simulations. J Comput Graph Stat. 1998;7:434–55.

6. Elangovan N, Lim J. Covid-19: S’porean students abroad heading home after government advisory but a few plan to stay put [Internet]. TODAY. 2020 [cited 2020 Apr 11]. Available from: https://www.todayonline.com/singapore/covid-19-sporean-students-overseas-prepare-head-home-after-government-advisory-few-vow

7. Cori A, Ferguson NM, Fraser C, Cauchemez S. A New Framework and Software to Estimate Time-Varying Reproduction Numbers During Epidemics. Am J Epidemiol [Internet]. 2013 Nov 1;178(9):1505–12. Available from: https://academic.oup.com/aje/article-lookup/doi/10.1093/aje/kwt133

8. Nishiura H, Linton NM, Akhmetzhanov AR. Serial interval of novel coronavirus (COVID-19) infections. Int J Infect Dis [Internet]. 2020 Apr;93:284–6. Available from: https://linkinghub.elsevier.com/retrieve/pii/S1201971220301193

9. Endo A, Abbott S, Kucharski AJ, Funk S. Estimating the overdispersion in COVID-19 transmission using outbreak sizes outside China. Wellcome Open Res [Internet]. 2020 Jul 10;5:67. Available from: https://wellcomeopenresearch.org/articles/5-67/v3

10. Bi Q, Wu Y, Mei S, Ye C, Zou X, Zhang Z, et al. Epidemiology and transmission of COVID-19 in 391 cases and 1286 of their close contacts in Shenzhen, China: a retrospective cohort study. Lancet Infect Dis [Internet]. 2020 Apr; Available from: https://linkinghub.elsevier.com/retrieve/pii/S1473309920302875

11. Churcher TS, Cohen JM, Novotny J, Ntshalintshali N, Kunene S, Cauchemez S. Measuring the path toward malaria elimination. Science (80- ) [Internet]. 2014 Jun 13;344(6189):1230–2. Available from: http://www.sciencemag.org/cgi/doi/10.1126/science.1251449
